# Anti-Inflammatory Activity of *Odina wodier* Roxb, an Indian Folk Remedy, through Inhibition of Toll-Like Receptor 4 Signaling Pathway

**DOI:** 10.1371/journal.pone.0104939

**Published:** 2014-08-25

**Authors:** Durbadal Ojha, Hemanta Mukherjee, Supriya Mondal, Aditya Jena, Ved Prakash Dwivedi, Keshab C. Mondal, Bharti Malhotra, Amalesh Samanta, Debprasad Chattopadhyay

**Affiliations:** 1 ICMR Virus Unit, I.D. & B.G. Hospital, Beliaghata, Kolkata, India; 2 Division of Microbiology, Department of Pharmaceutical Technology, Jadavpur University, Kolkata, India; 3 Department of Microbiology, Vidyasagar University, Midnapur, West Bengal, India; 4 Department of Microbiology, SMS Medical College & Hospital, Jaipur, India; National Center for Cell Science, India

## Abstract

Inflammation is part of self-limiting non-specific immune response, which occurs during bodily injury. In some disorders the inflammatory process becomes continuous, leading to the development of chronic inflammatory diseases including cardiovascular diseases, diabetes, cancer etc. Several Indian tribes used the bark of *Odina wodier* (OWB) for treating inflammatory disorders. Thus, we have evaluated the immunotherapeutic potential of OWB methanol extract and its major constituent chlorogenic acid (CA), using three popular *in vivo* antiinflammatory models: Carrageenan- and Dextran-induced paw edema, Cotton pellet granuloma, and Acetic acid-induced vascular permeability. To elucidate the possible anti-inflammatory mechanism of action we determine the level of major inflammatory mediators (NO, iNOS, COX-2-dependent prostaglandin E2 or PGE2), and pro-inflammatory cytokines (TNF-α, IL-1β, IL-6, and IL-12). Further, we determine the toll-like receptor 4 (TLR4), Myeloid differentiation primary response gene 88 (MyD88), c-Jun N-terminal kinases (JNK), nuclear factor kappa-B cells (NF-κB), and NF-kB inhibitor alpha (IK-Bα) by protein and mRNA expression, and Western blot analysis in drug treated LPS-induced murine macrophage model. Moreover, we determined the acute and sub-acute toxicity of OWB extract in BALB/c mice. Our study demonstrated a significant anti-inflammatory activity of OWB extract and CA along with the inhibition of TNF-α, IL-1β, IL-6 and IL-12 expressions. Further, the expression of TLR4, NF-κBp65, MyD88, iNOS and COX-2 molecules were reduced in drug-treated groups, but not in the LPS-stimulated untreated or control groups, Thus, our results collectively indicated that the OWB extract and CA can efficiently inhibit inflammation through the down regulation of TLR4/MyD88/NF-kB signaling pathway.

## Introduction

Most of the synthetic anti-inflammatory drugs are costly, and have adverse effect including gastrointestinal and respiratory irritation, nephrotoxicity, physical dependence and constipation in long-term use. Therefore, scientists are looking for cost-effective natural agents with low toxicity and better tolerance. The ethnomedicinal plants are considered as an important source of candidate therapeutics [1], [2], to combat long-term toxicity and escalating costs. Inflammation is a complex biological response of the damaged vascular tissues with protective attempt of healing, and classified as acute or chronic. The acute inflammation is the initial response of the body to the harmful stimuli, when increased movement of plasma and granulocytes takes place from blood to the injured tissues [3]; followed by a cascade of events involving the propagation and maturation of vascular and immune system, along with the cells of the injured tissues [4]. The affected cells are then activated to release several mediators including eicosanoids, cytokines, and chemokines to elicit the inflammatory response from acute to the chronic phase [1], [4]. In chronic (prolonged) inflammation, a progressive shift of injured cells occurred at site with simultaneous destruction and healing of the injured tissues [5], along with the release of cyclooxigenase (COX)-mediated prostaglandins (PGs), leading to the pain, edema and fever. Thus, COX inhibitors are used as antiinflammatory drugs. However, many COX inhibitors have serious adverse effects [6] and conventional nonsteroidal anti-inflammatory drugs (NSAD) are unsuitable for the management of chronic and silent inflammations [6].

Toll-like receptors (TLRs) are known to recognize pathogen-associated molecular patterns and induce innate immune responses; while lipopolysaccharides (LPS) of bacteria potently activate the dendritic cells (DCs) and monocyte/macrophages [7]. It is reported that LPS is recognized by TLR4 and induces the vigorous productions of various cytokines [7], [8]. Like other TLRs, the cytoplasmic tail of TLR4 contains a Toll-IL-1R (TIR) domain [9], [10], which upon activation recruits several TIR-containing intracellular adaptor proteins, including myeloid differentiate primary-response gene 88 (MyD88) [11], [12] and TIR domain-containing adaptor inducing IFN-β (TRIF) [13]. The MyD88-dependent signaling pathway activates mitogen-activated protein kinases (MAPKs) and nuclear factor-kappa B (NF-kB) to induces the inflammatory cytokines [12]. Recent reports showed that the phytochemicals of higher plants have therapeutic potential in inflammatory diseases [1], [14]–[20]. The primary health care system of many countries, including India, partly depends on traditional medicaments. One such medicament, *Odina wodier* Roxb. (Anacardiaceae) is a tall tree of tropical forests of Indian subcontinent, known as Rhus olina. Different part of this folk medicine is used for diverse ailments. The bark of the plant is used for gout, rheumatism, heart diseases, elephantiasis, ulcer, skin infection, wound healing [21], and as toothpowder. While the leaf juice is used to prevent abnormal white, clumpy discharge in women [22]; and the gum odina as tablet binder and emulsifier [23]. However, till date there is no scientific validation of such claims with proper pharmacological and phytochemical studies, except its anti-infective potential by this group [24].

In the present study we have analyzed the immunotherapeutic potential of the methanol extract of *Odina wodier* bark (OWB) against *in vivo* carrageenan- (acute) and dextran-induced (sub-acute) paw edema, cotton pellet granuloma (chronic), acetic acid induced vascular permeability, and the *in vitro* protein denaturation, to correlates its traditional use. Moreover, the antiinflammatory activity of chlorogenic acid (CA), a major component of OWB, was tested by acetic acid induced vascular permeability. Further, we have studied its immunomodulatory potential in murine macrophage model through the regulation of COX-2-dependent prostaglandin-E2 (PGE_2_) and TLR4 signaling pathways, including the p38 MAPK, JNK and NF-kB. Our results showed that OWB and CA treatment could restrict the inflammation in mice, and its anti-inflammatory effect is due to the down-regulation of TLR4/MyD88/NF-kB signaling pathway. Furthermore, the acute and sub-acute toxicity studies in Balb/C mice showed that the OWB bark extract is safe at its antiinflammatory dosage.

## Materials and Methods

### Materials

RPMI 1640, penicillin and streptomycin were obtained from Sigma Chemical Co. (St Louis, MO, USA); while fetal calf serum (FCS) from the Gibco BRL (Grand Island, NY, USA). The ELISA antibody kits (IL-1α, IL-6, IL-12 and TNF-α) were purchased from BD Biosciences (San Diego, CA); and antibodies against IkB-α, p-IKB-α NF-κB/p65, p38, p-p38 and GAPDH from Santa Cruz Biotechnology (Santa Cruz, CA). Tissue culture reagents were procured from the Gibco Laboratories, USA; while Primers for semi-quantitative PCR was from IDT, California, USA. Other chemicals were obtained either from the Sigma (USA) or the Merck (Germany).

### Collection, extraction and HPLC analysis of plant material

The collection, extraction, phytochemical analysis and HPLC purification of OWB was described previously [24]. Briefly, the *Odina wodier* stem bark, collected from Paschim Medinipur, West Bengal, India, was powdered (500 g), extracted in 95% methanol, and solvent evaporated in a Eyela Rotary Evaporator (Japan) at 40–45°C with an yield (w/w) of 9.34±0.46%. A weighted amount of extract was then dissolved in 0.1% (w/v) dimethyl sulfoxide (DMSO) and diluted in sterile distilled water for evaluation. The HPLC was done by a Shimadzu liquid chromatography system, consisting of binary LC-20AD pumps, coupled with a SPD-M20A photo-diode array detector, a Rheodyne 7725 injector with 20 µl loop, and a reverse phase C18 (250×4.6 mm, 5 µm) Zorbax column (Agilent, USA) at ambient (25°C) conditions. The mobile phase was 1% acetic acid in HPLC grade water: methanol (30∶70) with flow rate of 1 ml/min, and the absorbance was read at 210 nm. The stock solutions of OWB and chlorogenic acid (CA; Sigma, USA) was prepared by dissolving 10 mg of extract or CA in 10 ml of methanol separately, filtered (0.45 µm), and 20 µl of the sample was injected into the HPLC system. Identification of CA was based on their retention time and the comparison of their UV spectra. Quantitative analysis was achieved by five point calibration curves for CA, at 5–1000 µg/ml and from the equation Y = 6973.2 X+60941, where Y represents the area of the extract and X was the concentration of CA. The regression coefficient value of CA was 0.9955. Real samples were diluted accordingly to fit the dynamic linear range of the regression line, when necessary, and all measurements were performed in triplicates.

### Experimental animal

The animal (non-human primates) used were the female and male BALB/c mice (18–20 gm) and adult Wister rats (150–180 gm), acclimatized for 15 days in polypropylene cages in the Animal House facility, with standard food and water *ad libitum*. The animal experiments were conducted in accordance with the OECD guidelines, accepted by the Committee for the Purpose of Control and Supervision on Experiments on Animals (CPCSEA), Thiruvanmiyur, Chennai, India, and as per the approval of the Institutional Animal Care and Use Committee (IACUC) of Jadavpur University, Kolkata (Approval No: 0367/01/C/CPCSEA). When required, the surgical procedures performed under Ketamine hydrochloride (100 mg/kg i.m.) anesthesia, and all efforts were made to minimize the suffering of the animals.

### *In vivo* Toxicity study

Acute toxicity study was performed using different doses of OWB extract, administered orally to healthy 7-week-old BALB/c female mice, three times daily for 7 days. While for subacute toxicity study the animals were feed with the daily doses of the extract for 28 days. The control group (n = 10) received normal saline, while the experimental groups (six groups, n = 6) were administered with the text extract (0–3000 mg/kg body weight). The animals were observed continuously for 72 h and then daily upto 30 days to record any change in weight, behaviour, sign of clinical toxicity or morbidity, and to calculate the 50% lethality of the extract [25]. During the acute toxicity study, when required, cervical dislocation was used to euthanize animals. The criteria for euthanasia were (i) severe illness or the animals in a moribund state; (ii) severe pain and respiratory distress; (iii) abnormal vocalization, aggressiveness, posture and movements; (iv) self-induced trauma; (v) rapid weight loss, severe dehydration and significant bleeding. The mortality was calculated on the 30^th^ day, using weights and mortality data. Moreover, fresh blood was collected by cardiac puncture for the estimation of hematological and serum biochemical parameters, and finally the animals were sacrificed to collect liver, spleen and kidney for histopathological examination [17], [26].

### Carrageenan-induced rat paw edema

Inflammation in the right hind paw of each male Wistar rats (150–180 g), six per group, was made by subcutaneous (s.c.) injection of 0.1 ml of 1% carrageenan, under the sub-plantar aponeurosis [17], [18]. The animals of the test groups received 200 or 400 mg/kg of OWB extract orally, 1 h before the carrageenan injection; while the control group was received distilled water or 10 mg/kg Indomethacin, immediately after the carrageenan injection and at 1, 2, 3, 4 and 6 h. The paw volume was measured by a plethysmograph (up to the anatomical hairline on lateral malleolus) and compared with the controls. The inhibitory effect was calculated by the equation: Inhibition (%) = 100 – (edema volume in the treated/edema volume in the control) ×100.

### Dextran-induced rat paw edema

Following the above protocol 0.1 ml of dextran (1% w/v) in normal saline was used in the place of carrageenan [17].

### Cotton-pellet induced granuloma in rats

Sterile cotton-pellets (10 mg each) were implanted subcutaneously in the back axilla regions of each rat under Ketamine hydrochloride (100 mg/kg i.m.) anaesthesia. The animals in the two test groups were treated orally with OWB extract at 200 or 400 mg/kg daily for 7 consecutive days; while the control group received either normal saline (vehicle control) or Indomethacin (10 mg/kg body weight). Scarification was made after 8^th^ day and the cotton-pellets were removed, freed from extraneous tissues and the dry weight of each pellet was recorded. The percentage of inhibition with respect to the dry weight of the cotton-pellet was calculated and compared with the control groups [17], [18].

### Acetic acid-induced vascular permeability in mice

The effect of OWB extract and CA on the vascular permeability was tested in mice [27]. Randomly selected Balb/C mice were divided into six groups (n = 6): Group I was served as vehicle control, groups II and III received 200 and 400 mg/kg of OWB extract, group IV and V received 20 and 40 mg/kg of CA, while group VI was administrated with indomethacin (10 mg/kg), orally. One hour after the treatment, 200 µl of 0.2% Evan's blue in normal saline was injected through tail vein of each mouse (at 0.2 ml/20 g), and 30 min later each mouse was injected (i.p.) with 0.6% acetic acid in normal saline (0.2 ml). After 1h the animals were sacrificed, their abdomen was open to expose the entrails, and washed with normal saline (5 ml) to collect the content in a test tube. The content was then centrifuged, and the absorbance of the collected supernatant was measured in a spectrophotometer at 590 nm. The vascular permeability effects were expressed as the absorbance (A) of the amount of dye leaked into the intraperitoneal cavity [18].

### Effect on *in vitro* protein denaturation

Aliquots (1 ml) of OWB extract (50–200 µg/ml) or CA (5–20 µg/ml) or Indomethacin (10 µg/ml) was mixed with equal volume of egg albumin solution (1 mM) and incubated at 27±1°C for 15 min. The mixture was then placed at 70°C in a water bath for 10 min for denaturation of egg albumin, and the turbidity of the mixture was measured spectrophotometrically at 660 nm after cooling. Each experiment was carried out in triplicate to get the average; and the percentage inhibition of albumin denaturation was calculated by the formula: % inhibition of denaturation  =  (Abs of control – Abs of treated)/Abs of control X 100 [18], [27].

### Preparation of mouse peritoneal macrophages

Peritoneal macrophages from thioglycolated BALB/c mice were cultured for 48 h, as described previously [28].

### Cytotoxicity assay

Cytotoxicity was tested by MTT assay using macrophage cell monolayers, cultured in 96-well plates with RPMI 1640 and 10% FCS. The cell monolayer was incubated with OWB extract (0–1000 µg/ml) and CA (0–400) for 48 h, as described previously [24], [28].

### Measurement of Nitric Oxide, Cytokine and PGE2 release

Macrophage culture in 24 well plates (1×10^6^ cells/well) were induced with LPS (1 µg/ml), and incubated 1 h. The activated cells were then treated with the OWB extract (50 and 100 µg/ml) or CA (10 µg/ml) or LPS, and re-incubated for 24 h. The supernatant was removed and the concentration of NO was determined by Griess reagent (Sigma, USA) [17]; while the concentration of PGE2 and Cytokines were analyzed by sandwich ELISA, using commercial kit (BD Biosciences), as per manufacturer's instructions [18], [28].

### Isolation of RNA and real-time PCR

Macrophages plated in 12 well plate (4×10^6^ cells/well), were incubated with LPS (1 µg/ml) for 1 h. The attached cells were treated with the OWB extract (50 and 100 µg/ml) or CA (10 µg/ml) for 24 h. The RNA was isolated immediately using RNeasy Mini kit (QIAGEN), following the manufacturers protocol. Then the total RNA (0.1 mg/ml) in RNase-free water was mixed in 20 µl of RT mix (containing 5X VILO Reaction Mix, 10X SuperScript Enzyme Mix and DEPC treated water) and subjected to cDNA synthesis using the GeneAmp PCR System 9600 (Perkin Elmer Corp, USA). The real-time PCR was performed by using SYBR Green PCR Master Mix (Qiagen) following manufacturer protocol in an ABI Prism 7000 sequence detection system (Applied Biosystems, CA, USA). The PCRs were amplified at cycling conditions of: 95°C for 10 min and 40 cycles (15 s at 95°C, then 60 s at 60°C) in triplicate [28] using β-Actin as standard. Sequences of the PCR primers are listed in **Table 1**.

**Table 1:** Primer used in Real time-PCR assay.

Gene	Primer sequence
iNOS	Forward5′-CAAAGTCAAATCCTACCAAAGTTGACCTG-3′
	Reverse5′- TGCTACAGTTCCGAGCGTCAAAGAACCTG -3′
COX-2	Forward5′-GGAGAGACTATCAAGATAGTGATC-3′
	Reverse5′-ATGGTCAGTAGACTTTTACAGCTC-3′
IL-1β	Forward5′-TCATGGGATGATGATGATAACCTGCT-3′
	Reverse5′- CCCATACTTTAGGAAGACAGGGATTT-3′
IL-6	Forward5′-CTTCCAGCCAGTTGCCTTCTTG-3′
	Reverse5′-TGGTCTGTTGTGGGTGGTATCC-3′
IL-12	Forward5′- CCACTCACATCTGCTGCTCAACAAG -3′
	Reverse5′- ACTTCTCATAGTCCCTTTGGTCCAG -3′
TNF-α	Forward5′- GGCAGGTCTACTTTGGAGTCATTGC -3′
	Reverse5′- ACATTCGAGGCTCCAGTGAATTCGG -3′
MyD88	Forward5′- TACATACGCAACCAGCAG-3′
	Reverse5′- GGCAGTAGCAGATGAAGG -3′
TLR4	Forward5′-AGTGGGTCAAGGAACAGAAGCA-3′
	Reverse5′- CTTTACCAGCTCATTTCTCACC-3′
β- Actin	Forward5′- CCCACTCCTAAGAGGAGGATG -3′
	Reverse5′- AGGGAGACCAAAGCCTTCAT -3′

### Densitometry analysis

The respective RNA bands were analysed using a model GS-700 Imaging Densitometer and Molecular Analyst software (version 1.5; Bio-Rad Laboratories, Hercules, CA, USA).

### Western blot analysis

The LPS (1 µg/ml) induced peritoneal macrophage(s) were treated with the OWB extract (100 µg/ml) or CA (10 µg/ml) for 24 h. Then the equal amounts of extracted protein (40 µg/sample) from the whole cell were harvested in buffer (200 µl/well) containing 20 mM Tris (pH 7±0.5), 50 mM NaCl, 5% NP-40 and 0.05% DOC. The soluble fraction was then separated by centrifugation (16000 g for 10 min) at 4°C, subjected to SDS-PAGE and blotted to pre-equilibrated PVDF membrane (Thermo Scientific, USA). The membrane was then blocked in 5% NFDM in 1X TBST (20 mM Tris, pH 7.5, 150 mM NaCl, 0.5% Tween 20), rinsed and incubated with specific antibody in 5% BSA at 4°C overnight. Immunoblotting was performed with peroxidase-labelled specific antibodies and visualized by ECL Western blot detection kit (Millipore, USA) [29].

### Statistical analysis

Results were expressed as SEM (*n* = 6) and the statistical analysis were performed with one-way analysis of variance (ANOVA), followed by Dunnett's test. A value of *p*<0.05 was considered to be statistically significant, compared with the respective control.

## Results

### Standardization and physicochemical study of extract

The physiochemical study revealed that the OWB extract have the minimum content of water, total ash and acid insoluble ash, with maximum yield % (**Table S1 in File S1**), and the powdered extract was reddish to light magenta colour under visible to UV light. The preliminary phytochemical tests of OWB extract showed the presence of tannins, flavonoids, phenols, glycosides, saponins and phytosterols (**Table S2 in File S1**). The quantification of CA in HPLC chromatograms of standard and OWB extract samples (**Fig. S1 in File S1**) showed that the amount of CA, a pseudotannin, in the extract sample was 0.33% (w/w). We used CA as marker compound (standard) for the quantification of OWB extract, because tannin was found to be the major groups in the phytochemical test, and the related compounds are common in medicinal plants.

### *In vivo* Toxicity Studies

Acute toxicity studies revealed that the OWB extract was safe up to 1600 mg/kg body weight without any obvious toxicity in mice, and the 50% lethality was 1994.5 mg/kg body weight. Moreover, sub-acute toxicity studies revealed the normal haematological and biochemical profile including SGOT and SGPT level (**Table S3 in File S1**); and the histopathology of the kidney, liver and spleen of the extract treated mice were almost normal up to 28 days of treatment (**Fig. S2 in File S1**).

### Carrageenan-induced paw edema in rats

**Figure 1:**
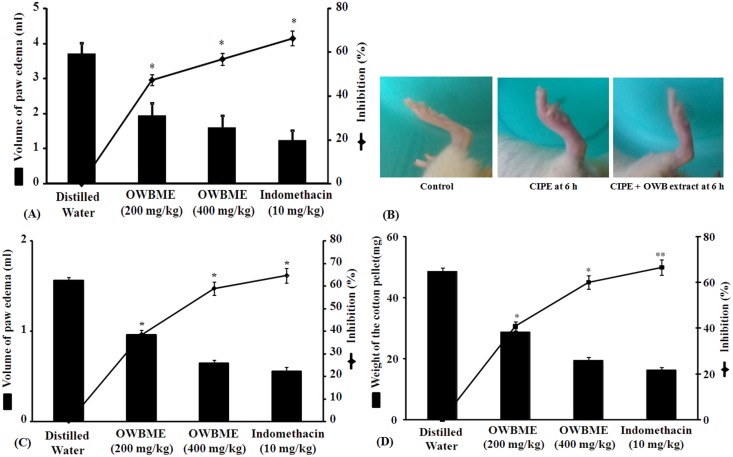
Effect of OWB extract on Carrageenan- and Dextran- induced paw edema, and Cotton pellet-induced granuloma in rats. (A and C) Inflammation in the right hind paw of Wistar rats was made by subcutaneous injection of carrageenan or dextran respectively, under the sub plantar aponeurosis. The test groups were administered with 200 or 400 mg kg^−1^ of OWB extract orally, 1 h before carrageenan or dextran injection; while the control group received distilled water or Indomethacin (10 mg/kg). After 6 h, the paw volume was measured and % of inhibition was compared with the control groups. (B) Photographs showing Carrageenan-induced Paw edema (CIPE) in the hind limb of rats, 6 h after carrageenan challenge. Redness and swelling of paw are evident with respect to control and OWB extract treatment. (D) Subcutaneously implemented sterile cotton-pellets (10 mg each) in the axilla regions of the rats, under anesthesia, were treated orally with the extract (200 or 400 mg kg^−1^) daily for 7 consecutive days, with respect to the normal saline or Indomethacin (10 mg/kg). After scarification, on 8^th^ day, the cotton-pellets were removed, cleaned and the dry weight of each pellet was taken to calculate the percentage of inhibition with respect to the cotton-pellet weight, compared with the control. Results are expressed as Mean ± SD, (n = 6), *, *P*<0.05, **, *P*<0.01.

The effect of OWB extract on the carrageenan-induced rat paw edema, presented in **Table S4 in File S1**, showed that the extract at 400 mg/kg inhibited 56.75% of paw edema, which is highly significant (P<0.01), compared to the animals treated with the standard drug indomethacin. On the otherhand, the extract at 200 mg/kg inhibits 47.29% of paw edema. Interestingly, the inhibition of edema volume (66.21%) by indomethacin (10 mg/kg) was nearly same with the OWB extract at 400 mg/kg (56.75%) after 6 h of treatment (**Fig. 1A**).

### Effect of dextran induced paw edema

In this model the maximum inhibition (64.74%) of paw edema swelling was noted with indomethacin (10 mg/kg), followed by OWB extract at 400 mg/kg (58.97%) and 200 mg/kg (38.46%), respectively. These data are significant (P<0.01) with respect to the controls, indicating the possible antiinflammatory activity of the OWB extract (**Fig. 1B**).

### Cotton pellet granuloma in rats

The results revealed that the oral treatment of OWB extract significantly inhibited both the exudatory and granulatory phase of inflammation, as the extract at 400 mg/kg showed 60.1% inhibition of granuloma, nearly similar to the inhibition (66.58%) produced by indomethacin (**Fig. 1C**).

### Acetic acid-induced vascular permeability

The results of the acetic acid-induced vascular permeability, presented in **Table 2**, showed that the oral pretreatment of OWB extract (200 and 400 mg/kg) produced 37.30 and 58.75% inhibition; while the CA (20 and 40 mg/kg) showed 49.46 and 62.98% inhibition, but Indomethacin (10 mg/kg) inhibited 64.73% of peritoneal protein exudation. Here, the inhibition of vascular permeability by OWB extract at 400 mg/kg is significant (p<0.01) compared to the indomethacin group (p<0.001).

**Table 2:** Effect of OWB extract on acetic acid-induced vascular permeability.

Treatment	Dose (mg/kg)	Concentration of protein (mg/ml)	Inhibition (%)
Vehicle	—	24.1±0.24	0
Indomethacin	10	8.5±0.26	64.73±0.33*
OWB extract	200	15.11±0.14	37.30±0.44**
	400	9.94±0.18	58.75±0.24**
CA	20	12.18±0.33	49.46±0.55*
	40	8.92±0.28	62.98±0.33**

Values plotted as mean ± SEM; n = 6) * p<0.001** p<0.01.

### Inhibition of Protein denaturation

The inhibitory effect of OWB extract on protein denaturation, presented in **Table 3**, showed that the OWB extract at 50–200 µg/ml and CA at 5–20 µg/ml had significant (p<0.001) inhibition (37.94–70% and 39.41–71.17%) of egg albumin denaturation, compared to indomethacin (74.71%) at 100 µg/ml.

**Table 3:** Effect of OWB extract and CA on protein denaturation.

Sample(s)	Concentration (µg/ml)	Absorption at 660 nm	% inhibition of protein denaturation
Vehicle Control	-	0.680±0.08	-
OWB extract	50	0.422±0.13	37.94±0.32*
	100	0.236±0.35	65.29±0.55*
	200	0.204±0.23	70.00±0.66*
CA	5	0.412±0.062	39.41±0.12**
	10	0.225±0.15	66.91±0.16*
	20	0.196±0.2	71.17±0.66*
Indomethacin	100	0.172±0.17	74.71±0.33*

Each value represents the Mean ± SD; n = 6) * p<0.001** p<0.01.

### Cytotoxicity assay

The MTT assay revealed that the 50% cytotoxicity (CC_50_) of OWB extract and CA on macrophage cell monolayer was 386.6 and 182.5 µg/ml, respectively.

### NO generation and iNOS2 expression in LPS-induced murine peritoneal macrophages

**Figure 2:**
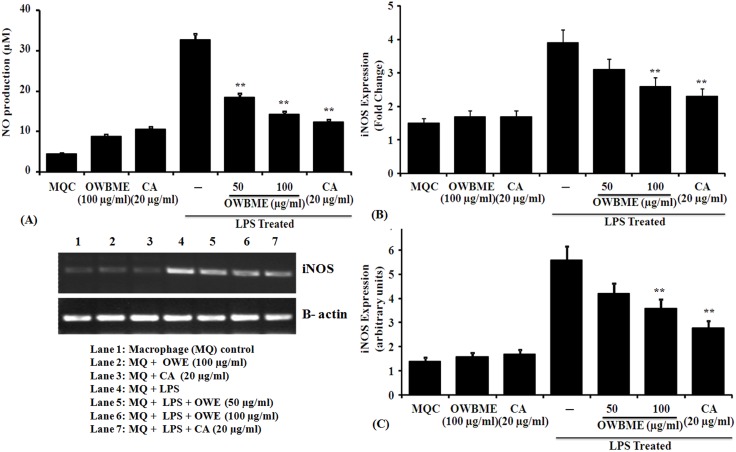
Immunomodulatory activity of OWB extract and CA via nitrite generation and iNOS2 expression in LPS-induced murine macrophages. (A) Macrophages (10^6^ cells/ml) were incubated with LPS (1 µg/ml), OWB (50 and 100 µg/ml) or CA (10 µg/ml) for 24 h. The cell-free supernatants were collected for nitrite assay, as described in the Materials and methods. Data were expressed as Means ± SD from triplicate experiments, yielding similar results (µ moles of nitrite). Asterisks indicate a statistically significant increase (**P, 0.05) in nitrite generation, compared to the infected macrophages. (B–C) The LPS-stimulated macrophages were treated with the OWB extract and CA, incubated for 24 h, following which RNA was isolated and subjected to the RT–PCR analysis for the expression of iNOS2 mRNA. The data were expressed as Mean ± SD from triplicate experiments yielding similar results. The asterisk indicates a statistically significant increase (**P, 0.001) in iNOS2 expression, compared to the infected macrophages.

To study whether the OWB extract and CA modulates nitrite (NO) production, we determined the release of NO in normal and LPS-stimulated macrophages. The results showed that the NO production in activated macrophages was significantly reduced in OWB extract and CA treated groups, compared to the LPS-induced macrophage control (**Fig. 2A**). Further, to confirm the involvement of NO, we analyze the mRNA expression of drug-treated macrophage by RT–PCR, and found that the OWB extract and CA treatment caused a significant reversal of the iNOS2 inhibition in LPS-induced macrophages (**Fig. 2B and 2C**).

### Effect of OWB extract and CA on COX-2-mediated PGE2 release in macrophages

**Figure 3:**
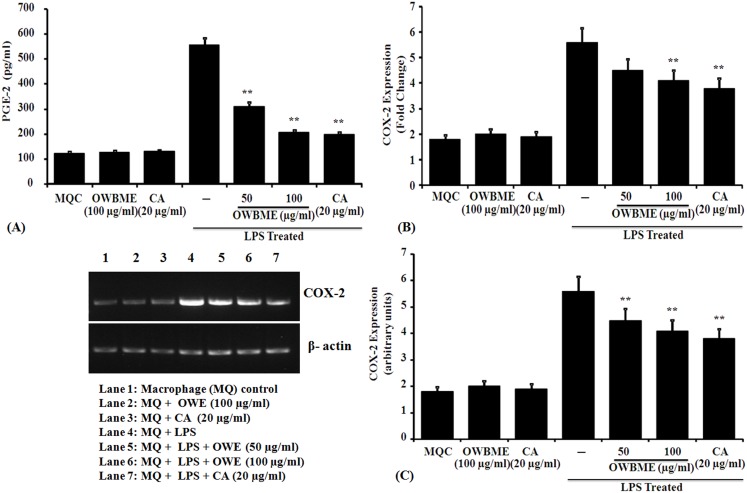
Effect of OWB extract and CA on PGE2 release and COX-2 expression in LPS-induced murine peritoneal macrophages. (A) Overnight macrophage cultures were stimulated with LPS (1 µg/ml), and treated with OWB (50 or 100 µg/ml) or CA (10 µg/ml). After 24 h the treated cells were used to determine the PGE-2 (pg/ml) level by ELISA. PGE2 values represent the Means ± SD from four different experiments. The asterisk indicates statistically significant decrease (**P, 0.001) in PGE2 levels, compared with LPS stimulated macrophages. (B–C) Similarly treated cells, incubated for 24 h, were used for the isolation of RNA, and the isolated RNA was subjected to RT–PCR analysis for COX-2 mRNA. The RT–PCR data are expressed as Means ± SD from triplicate experiments. Asterisks indicate a statistically significant decrease (**P, 0.05) in COX-2 expression, compared with infected macrophages.

To elucidate the protective mechanism of OWB extract and CA against inflammatory conditions we have investigated whether the extract and CA regulates PGE2 release in LPS-induced macrophages. The results revealed that the PGE-2 production was considerably higher in LPS-induced macrophage, compared to the macrophage control; while the OWB and CA-treated cells had a significantly reduced PGE2 level (**Fig. 3A**). Therefore, we further assessed the expression of COX-2, the rate-limiting enzyme in PGE2 biosynthesis, at the mRNA levels, and observed an increased COX-2 expression in stimulated macrophages, while the OWB or CA treatment significantly inhibited its expression at mRNA level (**Fig. 3B and 3C**).

### Effects of OWB extract and CA on TLR4 signaling pathway

**Figure 4:**
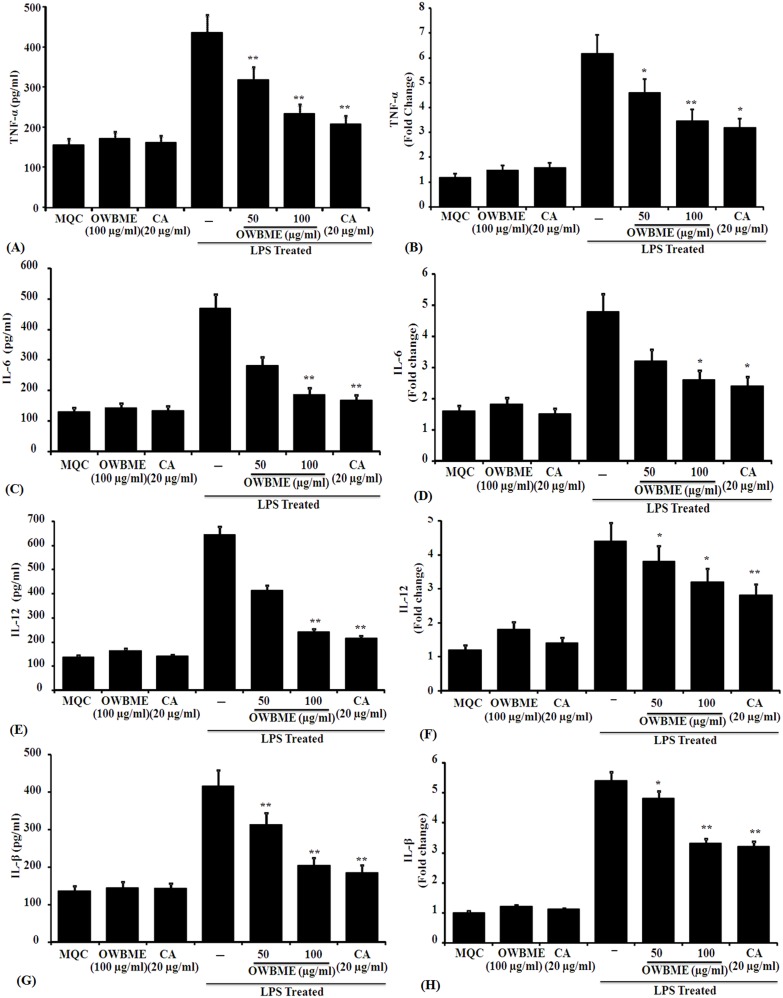
Effect of OWB extract and CA on pro-inflammatory and anti-inflammatory cytokine release in LPS-induced peritoneal macrophages by sandwich ELISA and RT-PCR. Peritoneal macrophages were cultured overnight and incubated with LPS (1 µg/ml), washed after 4 h and then treated with the OWB (50 or 100 µg/m) or CA (10 µg/ml). The cells were further incubated for 24 h, and the cell-free supernatants were subjected to sandwich ELISA to determine the level of (A) TNF-α, (C) IL-1β, (E) IL-6 and (G) IL-6 (pg/mL). In a separate set similarly treated cells were cultured for 5 h, and collected in TRI Reagent for mRNA extraction and subsequent RT–PCR analysis (vide Materials and methods) to study the cytokine and β-actin mRNA expression. The data were shown for the expression of (B) TNF-α, (D) IL-1β, (F) IL-6 and (H) IL-12. The ELISA and RT–PCR data are expressed as Means ± SD from triplicate experiments, yielding similar results. Asterisks indicate statistically significant (**P, 0.05) induction of TNF-α, IL-1β, IL-6, and IL-12 release; and increase or decrease (**P, 0.05; *P, 0.001) in cytokine expression, compared to the infected macrophages.

**Figure 5:**
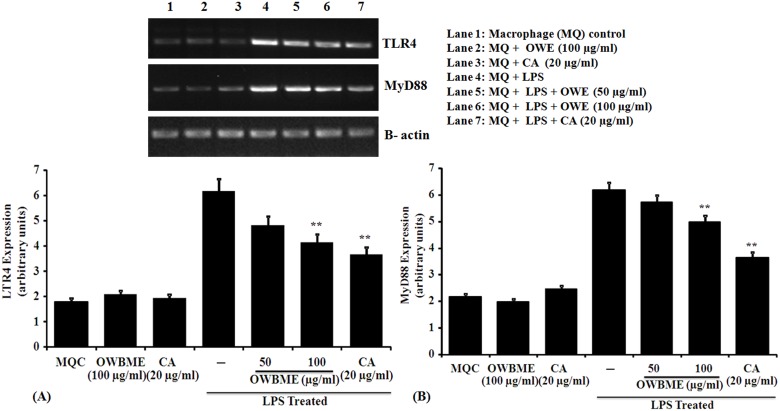
Effect of OWB extract and CA on TLR4 and MyD88 expression. The LPS-stimulated macrophages were treated with the OWB or CA and incubated for 24 h, following which RNA was isolated for RT–PCR analysis of the expression of MyD88 (A) and TLR4 (B) mRNA. The RT–PCR data are expressed as Means ± SD from triplicate experiments. The expression of TLR4 and MyD88 was significantly higher in the LPS-induced macrophage as compared to the control and OWB or CA co-treated group (** *P*<0.001).

**Figure 6:**
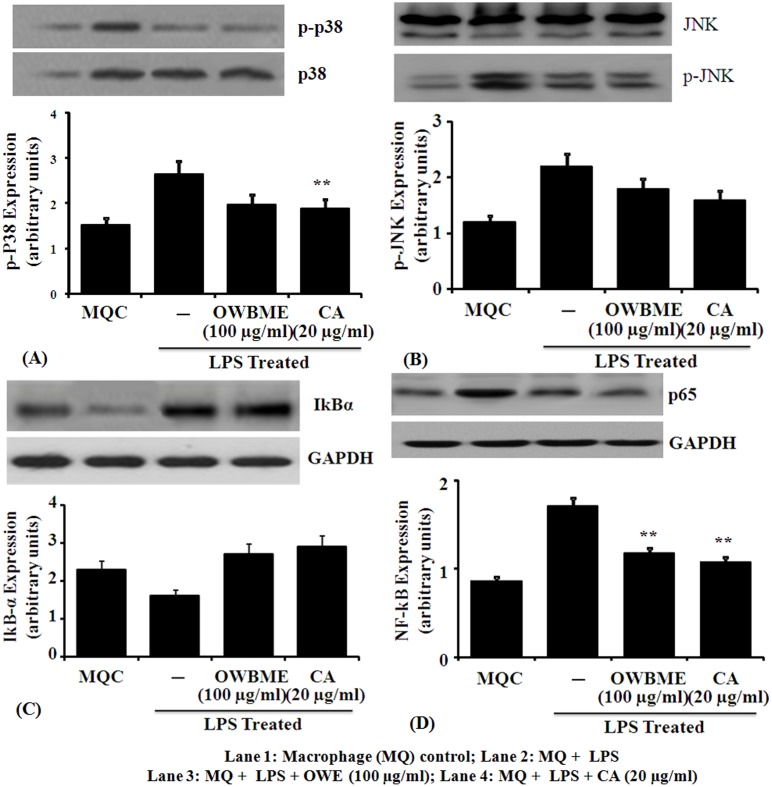
Effect of OWB extract and CA on the JNK, MAPK, IkB-α and MyD88 expression. Expression of (A) JNK, (B) MAPK, (C) IkB-α and (D) MyD88 were determined by the Western blot, using GAPDH as the internal control. The LPS (1 µg/ml) induced peritoneal macrophage(s) were treated with OWB (100 µg/ml) or CA (10 µg/ml), and after 24 h the protein extract from whole cell were harvested in buffer, containing 20 mM Tris (pH 7±0.5), 50 mM NaCl, 5% NP-40 and 0.05% DOC. The soluble fraction was separated by centrifugation, subjected to SDS-PAGE and blotted to pre-equilibrated PVDF membrane. The membrane was then blocked in 5% NFDM in 1X TBST, rinsed and incubated with specific antibody at 4°C overnight. Immunoblotting was performed with peroxidase-labelled specific antibodies and visualized by ECL Western blot detection kit. The average expression of NF-kB and MAPK was significantly higher in the LPS-induced macrophage, as compared to the control and OWB or CA co-treated group (** *P*<0.001).

The results presented in **Fig. 4** showed that the expression of proinflammatory cytokines TNF-α, IL-6, IL-12 and IL-1β were up-regulated in LPS-treated macrophage. As the TLR4 signaling pathway is known to play an important role in the progression of inflammation, so we have tested the effect of OWB extract and CA on TLR4 signaling pathway to elucidate the precise molecular mechanism of anti-inflammatory activity. It was observed that the mRNA levels of TLR4 and MyD88 (**Fig. 5**) were significantly increased after LPS treatment, compared to the control group. Moreover, the LPS treatment could reduce the nuclear translocation of NF-kB (**Fig. 6**). All these data collectively suggested that the treatment of OWB extract and CA activated the TLR4/MyD88/NF-kB signaling pathway Further, the results also revealed that the OWB and CA treatment leads to the inhibition of TLR4 signaling pathway activation, including the decreased expression of TLR4, MyD88, iNOS and COX-2; down regulation of TNF-α, IL-12, IL-6, IL-1β; and reduced translocation of NF-kB and p-P38 with increased IkBα protein.

## Discussion

In this study, we have validated the *in vivo* anti-inflammatory activity of the OWB extract by three popular models, along with the vascular permeability, and albumin denaturation. Moreover, we have established the underlying mechanism of anti-inflammatory action through the demonstration of activated TLR4 signaling pathway by the OWB extract and its major compound CA, including the toxicity profile of the extract. The OWB extract at 40 mg/kg inhibit 56.75% of carrageenan and 58.97% of dextran-induced paw edema (**Fig. 1**), compared to indomethacin, suggests the possible anti-inflammatory activity of the extract. The carrageenan-induced paw edema model was used due to its wide acceptability and biphasic sequential release of chemical mediators [18], where the vasoactive histamine and 5-HT were released in the early phase and the prostaglandins (kinin) in the acute late phase [30]. These mediators collectively results in the increased vascular permeability, leading to the accumulation of fluid in the tissues to form edema [31]. While dextran mediated inflammation is caused through both histamine and serotonin release [17]. The reduction of carrageenan and dextran-induced inflammation by OWB extract suggests its ability to block the release of any of those mediators. Moreover, the OWB extract at 400 mg/kg drastically inhibited (60.1%) the cotton-pellet induced granuloma, like the standard drug indomethacin (66.58%), indicating its anti-inflammatory activity, probably by reducing the number of fibroblasts with the synthesis of collagen and muco-polysaccharides during the granuloma tissue formation.

We have used the acetic acid-induced vascular permeability assay to confirm the anti-inflammatory potential of the test extract and found a significant inhibition (58.75% and 68.98%) of permeability at 400 mg/kg of OWB extract and 40 mg/kg of CA, respectively, compared to indomethacin (64.73%). It is known that the acetic acid, a vasodilator, dilate blood vessels to release histamine, prostaglandins and leukotrienes, leading to the increased vascular permeability, with an immediate sustained reaction [32]. Thus, the inhibition of vascular permeability by OWB suggests that its phytoconstituent may effectively suppress the exudative phase of acute inflammation. The denaturation of protein is a well-known cause of inflammation [33] and many anti-inflammatory drugs can inhibit thermally induced protein denaturation [15]–[18]. Here, the ability of OWB extract and CA to bring down the denaturation of protein is probably a contributing factor for its anti-inflammatory activity, due to the presence of flavonoids [34] and tannin [35] in the OWB extract, as observed with the phytochemical tests. The HPLC analysis of OWB extract showed the presence of chlorogenic acid as one of the active constituent, with known anti-inflammatory activity [19], [20]. Furthermore, the oral acute and subacute toxicity studies of OWB extract showed no mortality or clinical toxicity, or gross abnormality in the kidney, liver and spleen of the tested animals, indicating that the extract has good safety profile.

It is reported that in arachidonic acid metabolism PGE2, thromboxane A2, leukotrienes B4 and leukotrienes C4 have no role on NO induction [36]. Thus, we have investigated the production of PGE2, an oxygenated arachidonic acid metabolite, produced by the COX pathway. The PGE2 plays an important role in regulating the development of a response dominated by Th1- or Th2-associated lymphokines [37]; and its biosynthesis involves two consecutive rate-limiting reactions requiring COX enzyme, followed by specific PGE syntheses. In contrast to COX-1, the expression of COX-2 is rapidly induced by any harmful stimuli [38] and thus, plays an important role in the PGE2 production during many patho-physiological processes [39]. Following infection or injury, increased PGE2 is reported to influence the immune response by several mechanisms, including the inhibition of Th1 cytokines IL-2, IL-12 and IFN-γ [37]; impairing phagocytosis and lymphocyte proliferation [40]. Several studies have reported the inhibition of NO production by prostaglandins, especially PGE2, in macrophage model [36], [41]. The inhibition of PGE2 production by indomethacin is known to restore the NO levels within the macrophage in co-cultured LPS-stimulated hepatocytes [42]. This suggest a mechanism for macrophage-derived-PGE2-mediated NO suppression, and a rationale for the rapid induction of COX-2 and sustaining elevated levels of PGE2 during infection [42]. We observed that OWB extract and CA suppressed PGE2 synthesis by inhibiting COX-2, followed by concomitant inhibition of NO production due to decreased iNOS2 mRNA expression in LPS-stimulated macrophages (**Fig. 2** and **3**). Further, the OWB and CA treatment significantly reduced the expression of IL-1β, IL-6, TNF-α and IL-12 in LPS- stimulated macrophage (**Fig. 4**).

TLR4, a member of the TLR protein family, is essential for LPS-mediated signaling. LPS after binding to TLR4 triggers two critical intracellular signaling pathways, MyD88-dependent and MyD88-independent cascades [43]. The MyD88-dependent pathway signals through the activation of ikB kinase (IKK), which in turn activates the transcription factor NF-kB, and controls the expression of proinflammatory cytokines with other immune-related genes [12]. The rapidly increasing knowledge in the relationship between the TLR4 signaling and inflammatory disorders makes this signaling inhibition a promising strategy for the prevention of inflammatory diseases [10]. In inflammation, TLR4 and its ligand LPS, mediate their effects through different mechanisms [10]. In Kupffer cell, TLR4 activation produces pro-inflammatory cytokines TNF-α, IL-1β, IL-6 and IL-12, resulting in hepatocyte damage, leukocyte infiltration and secretion of pro-fibrogenic cytokines [44]. While, the activation of TLR4 enhances TGF-β signaling in hepatic stellate cells (HSCs) through the down-regulation of a TGF-β1 pseudo-receptor Bambi [45], and TLR4-MyD88-NF-kB dependent pathway, thereby sensitizing HSCs to TGF-β1 signaling [46]. Regulation of Bambi by TLR4 signaling provides a link between pro-inflammatory and profibrogenic signals. In addition, liver endothelial cells (LECs) can express TLR4 signaling to regulate angiogenesis through MyD88 pathway, linked to the development of liver fibrosis [47]. Moreover, the LPS elevated mRNA levels of TLR4 and MyD88 (**Fig. 5**) leads to the activation of NF-kB (**Fig. 6**) and thereby increasing the expression of proinflammatory cytokines *i*NOS (**Fig. 2**), COX-2 (**Fig. 3**), TNF-α, IL-6, and IL-1β (**Fig. 4**) in the macrophage. Taken together, our results suggest that the OWB extract and CA decreased the mRNA and protein expression of proinflammatory cytokines, including *i*NOS, COX-2, TNF-α, IL-6 and IL-1β, and their upstream signaling molecules TLR4 and MyD88. Moreover, the OWB and CA may reduce inflammation through the suppression of TLR4-mediated proinflammatory signaling cascades. Here, we have systematically evaluated how the existing murine models mimic human inflammatory diseases, and found that the used models need to be critically evaluated before accepting its relevance to the human diseases. Thus, further study may explore these results in a suitable human disease model.

## Conclusions

This study validated the traditional medicinal use of *Odina wodier* bark and its beneficial effect in the management of ailments related to the inflammatory diseases in primary health care. However, to know the exact mechanism of its anti-inflammatory activity further studies are required.

## Supporting Information

File S1 **Table S1, The result of physical studies of OWB extract. Table S2, The detection of the active constituent in OWB extract. Table S3, Hematological parameters of animals after 28 days oral treatment with OWB extract. Table S4, Effect of OWB extract on carrageenan induced paw edema in rats. Figure S1, (A) HPLC of Chlorogenic acid; (B) HPLC of methanol extract of *Odina wodier* Bark. Figure S2, Histology of organs from mice untreated or treated with OWB extract (1.6 g/kg b.w).** (A) Normal liver; (B) Normal spleen; (C) Normal kidney; (D) OWB extract treated liver; (E) OWB extract treated spleen; and (F) OWB extract treated kidney.
(DOC)
