# Correction: Anti-Inflammatory Activity of *Odina wodier* Roxb, an Indian Folk Remedy, through Inhibition of Toll-Like Receptor 4 Signaling Pathway

**DOI:** 10.1371/journal.pone.0288420

**Published:** 2023-07-07

**Authors:** Durbadal Ojha, Hemanta Mukherjee, Supriya Mondal, Aditya Jena, Ved Prakash Dwivedi, Keshab C. Mondal, Bharti Malhotra, Amalesh Samanta, Debprasad Chattopadhyay

In Fig 5, the agarose gel picture for TLR4 expression has been mistakenly replaced by Fig 2 iNOS expression depicting agarose gel. The densitometric estimations shown in Fig 5A correspond to the correct gel that was misplaced earlier. Please see the correct Fig 5 with TLR4 expression showing agarose gel here. In Table 1, the primers for MyD88 are incorrect. Please see the correct Table 1 here.

**Fig 5:**
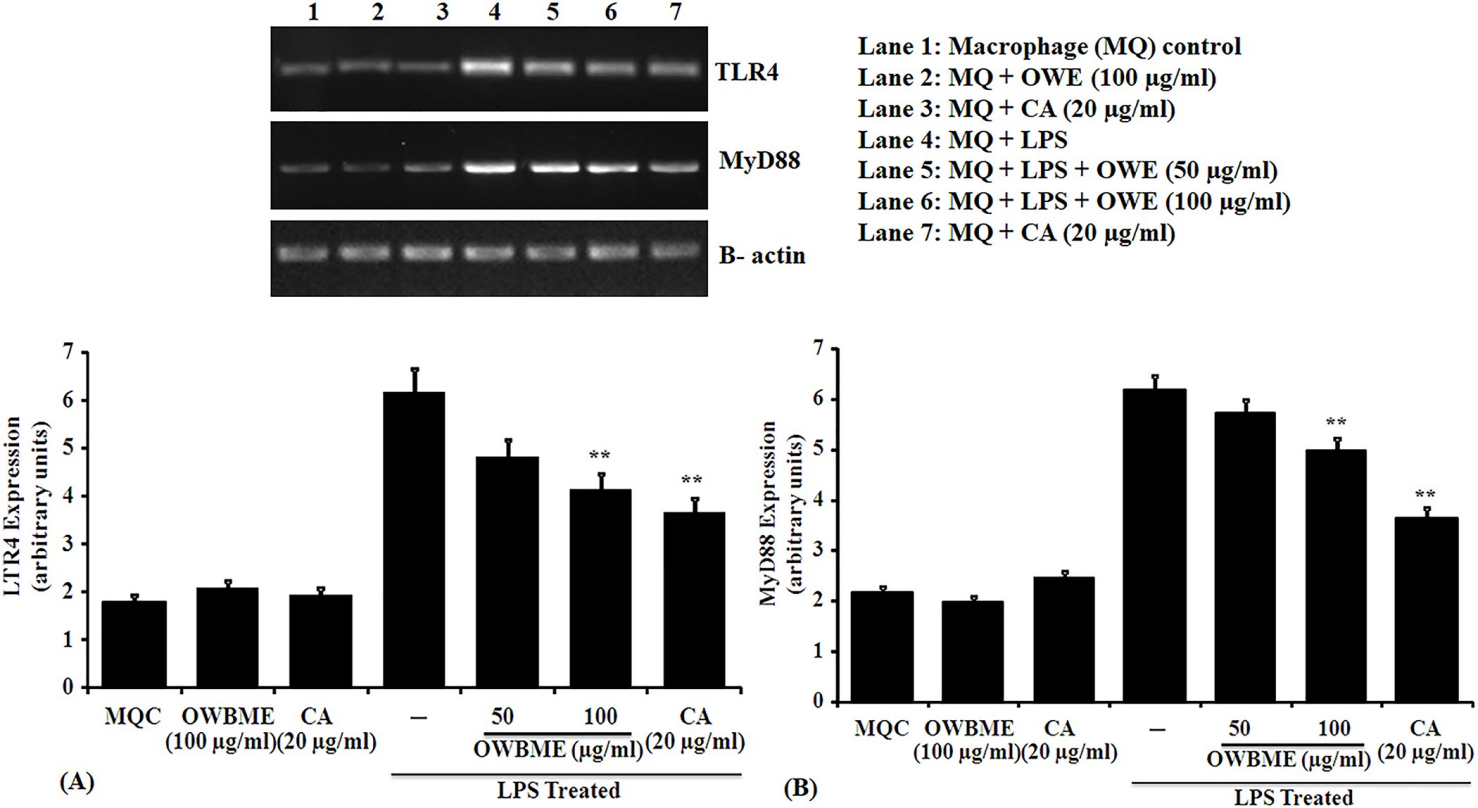
Effect of OWB extract and CA on TLR4 and MyD88 expression. The LPS-stimulated macrophages were treated with the OWB or CA and incubated for 24 h, following which RNA was isolated for RT–PCR analysis of the expression of MyD88 (A) and TLR4 (B) mRNA. The RT–PCR data are expressed as Means ± SD from triplicate experiments. The expression of TLR4 and MyD88 was significantly higher in the LPS-induced macrophage as compared to the control and OWB or CA co-treated group (** P<0.001).

**Table 1:** Primer used in Real time-PCR assay.

Gene	Primer sequence
iNOS	Forward5’-CAAAGTCAAATCCTACCAAAGTTGACCTG-3′
	Reverse5′-TGCTACAGTTCCGAGCGTCAAAGAACCTG-3′
COX-2	Forward5′-GGAGAGACTATCAAGATAGTGATC-3′
	Reverse5′-ATGGTCAGTAGACTTTTACAGCTC-3′
IL-1β	Forward5′-TCATGGGATGATGATGATAACCTGCT-3′
	Reverse5′- CCCATACTTTAGGAAGACAGGGATTT-3′
IL-6	Forward5′-CTTCCAGCCAGTTGCCTTCTTG-3′
	Reverse5’-TGGTCTGTTGTGGGTGGTATCC-3’
IL-12	Forward5′-CCACTCACATCTGCTGCTCAACAAG-3′
	Reverse5′-ACTTCTCATAGTCCCTTTGGTCCAG-3′
TNF-α	Forward5′-GGCAGGTCTACTTTGGAGTCATTGC-3′
	Reverse5′-ACATTCGAGGCTCCAGTGAATTCGG-3′
MyD88	Forward5′-CATGGTGGTGGTTGTTTCTG-3′
	Reverse5′-CTGTTGGACACCTGGAGACA-3′
TLR4	Forward5′-AGTGGGTCAAGGAACAGAAGCA-3′
	Reverse5′-CTTTACCAGCTCATTTCTCACC-3′
β- Actin	Forward5′-CCCACTCCTAAGAGGAGGATG-3′
	Reverse5′-AGGGAGACCAAAGCCTTCAT-3′
