# Supplementary material for: Anti-Inflammatory Activity of Odina wodier Roxb, an Indian Folk Remedy, through Inhibition of Toll-Like Receptor 4 Signaling Pathway
Source: PLoS One. 2014 Aug 25;9(8):e104939. doi: 10.1371/journal.pone.0104939 (PMC4143220; doi:10.1371/journal.pone.0104939)
Supplement: File S1 — Table S1, The result of physical studies of OWB extract. Table S2, The detection of the active constituent in OWB extract. Table S3, Hematological parameters of animals after 28 days oral treatment with OWB extract. Table S4, Effect of OWB extract on carrageenan induced paw edema in rats. Figure S1, (A) HPLC of Chlorogenic acid; (B) HPLC of methanol extract of Odina wodier Bark. Figure S2, Histology of organs from mice untreated or treated with OWB extract (1.6 g/kg b.w). (A) Normal liver; (B) Normal spleen; (C) Normal kidney; (D) OWB extract treated liver; (E) OWB extract treated spleen; and (F) OWB extract treated kidney. (DOC) [file pone.0104939.s001.doc]

**Supporting Information S1**

**Table S1**:

| Sample (g) | % Yield (w/w) of OWB extract | % rendement (yield) | Ash content  (% w/w) | Acid insoluble ash (% w/w) | Water content (%) |
| --- | --- | --- | --- | --- | --- |
| 600±0.5 | 9.34±0.46 | 12.1±0.51 | 4.55 ± 0.55 | 0.24 ± 0.33 | 24.69 ±0.50 |

**Table S2.**

| Extracts | Alkaloid | Carbohydrate | Glycoside | Saponin | Phytosterol | Phenol | Tannin | Flavonoid |
| --- | --- | --- | --- | --- | --- | --- | --- | --- |
| OWB extract | – | ++ | + | – | + | +++ | +++ | ++ |

* ‘–’ shows absent of phytochemical

*‘+’ shows present of phytochemical

**Table S3**.

| **Parameters** | **Control** | **Methanol extract** | |
| --- | --- | --- | --- |
|  |  | **800 mg/kg** | **1600 mg/kg** |
| Hb (g %) | 12.33 ± 0.03* | 12.16±0.012* | 12.56±0.024* |
| RBC (10^6^/Cu.Mm) | 3.59±0.008* | 3.48±0.032** | 3.56±0.01** |
| Total WBC (10^3^/Cu.Mm) | 8.16±0.052** | 8.34±0.074* | 8.08±0.013** |
| Clotting time (S) | 111.42±0.084* | 111.76±0.016** | 111.32±0.012* |
| Cholesterol | 93.96±0.54* | 94.66±0.66* | 93.08±0.50* |
| SGOT | 107.4±0.44** | 108.6±0.25* | 105.2±0.33** |
| SGPT | 28.86±0.33* | 29.34±0.75* | 27.66±0.44* |
| Alkaline Phosphatase (U/L) | 266.46±0.66* | 268.24±0.74** | 272.44±0.24* |
| Blood Glucose | 117.2±0.22** | 118.4±0.33* | 119.6±0.56** |

Results are expressed as Mean ± SEM, (n=6) *, *P*<0.05, **, *P*<0.01; Control, 2% (v/v) aqueous Tween-80 solution.

**Table S4**.

| Treatment | Dose (mg/kg) | Volume of paw edema (ml) at various time intervals. | | | | % of Inhibition at 6h |
| --- | --- | --- | --- | --- | --- | --- |
|  |  | 0h | 1h | 3h | 6h |  |
| Vehicle | -- | 1.35±0.12 | 2.30±0.16 | 3.10±0.2 | 3.70±0.28 | 0 |
| Indomethacin | 10 | 1.15±0.15* | 1.90±0.11** | 1.55±0.18* | 1.25±0.14* | 66.21±0.6* |
| OWB extract | 200 | 1.25±0.18* | 2.20±0.21* | 2.10±0.18* | 1.95±0.25* | 47.29±0.2* |
|  | 400 | 1.30±0.15** | 2.15±0.17** | 1.85±0.16* | 1.60±0.18* | 56.75±0.4* |

Results are expressed as Mean ± SEM, (n=6) *, *P*<0.05, **, *P*<0.01; Control, 2% (v/v) aqueous Tween-80 solution.

(A)


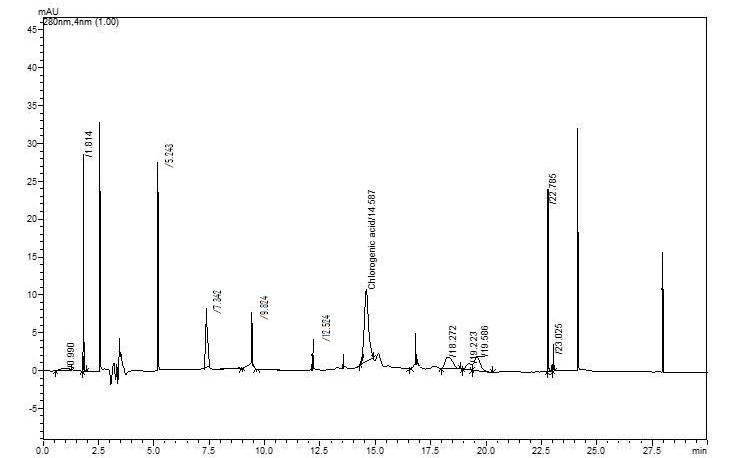


(B)

**Figure S1.**


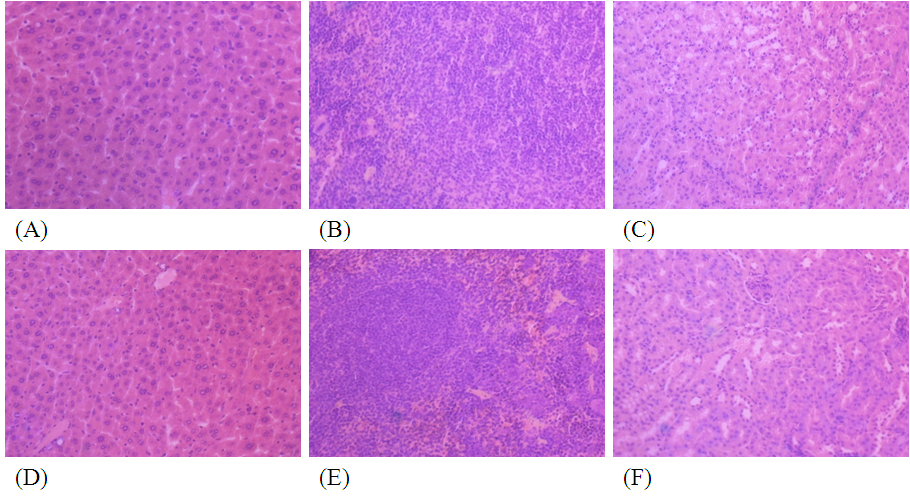


**Figure S2.**
